# Spontaneous breathing *vs*. mechanical ventilation for respiratory-gated MR-HIFU ablation in the liver

**DOI:** 10.1186/2050-5736-3-S1-O87

**Published:** 2015-06-30

**Authors:** Joost Wijlemans, Johanna van Breugel, Martijn de Greef, Chrit Moonen, Maurice van den Bosch, Mario Ries

**Affiliations:** 1 University Medical Center Utrecht, Utrecht, Netherlands

## Background/introduction

Magnetic resonance-guided High Intensity Focused Ultrasound (MR-HIFU) ablation in the liver is complicated by the continuous motion of the target due to the respiratory cycle. Several motion compensation strategies have been proposed in the past, such as breath-holding, respiratory gating and dynamic beam steering. Respiratory gating for sonication and MR thermometry uses a pencil beam navigator on the diaphragm to limit power output and image acquisition to the resting phase of the diaphragm.

Previously, we have used General Anesthesia with mechanical ventilation (GA) to obtain a long and reproducible resting phase of the diaphragm. From a patient’s perspective however, Procedural Sedation and Analgesia (PSA) has several advantages over GA such as a lower risk of complications and shorter recovery. In addition, it has lower associated costs and can be performed by non-anesthesiologists. The purpose of this animal study was to investigate the feasibility of respiratory-gated MR-HIFU ablation in the liver under PSA with spontaneous breathing.

## Methods

Normal Dalland land pigs (n = 2) were placed in prone position on a clinical Sonalleve MR-HIFU therapy system integrated with a 1.5T Achieva MRI (Philips Healthcare) with minor modifications. First, PSA was induced using continuous intravenous (i.v.) infusion of and propofol (4.5 mg/kg/h) and remifentanil (6,6 μg/kg/h). Remifentanil was chosen because of its depressant effect on the respiration combined with a short plasma half-life of <10 min. Volumetric sonications were performed under PSA (4 mm diameter trajectory, 450 W, 20 – 25 sec.). Subsequently the animal was intubated and GA was induced using continuous i.v. infusion of midazolam (1 mg/kg/h), cisatracurium (0.09 mg/kg/h), and sufentanil (11.3 mg/kg/h). Mechanical ventilation was set to 13/min (cycle: 4.5 sec, resting phase: 3 sec). A similar lesion was sonicated using the same sonication protocol. For both the GA and PSA experiments, the non-perfused volumes (NPVs) on contrast-enhanced imaging were expressed in milliliter and the duty cycles of the therapeutic sonications were expressed in median percentages (interquartile range, range) and compared using the Mann-Whitney U test.

## Results and conclusions

A total of 38 sonications were performed in two animals. Under GA, a median duty cycle of 65.0% (IQR 62.6 - 66.5, range 46.0 - 68.7) was achieved. Under PSA, a duty cycle of 80.0% (IQR 77.5 - 85.6, range 18.0 - 89.9) was achieved (p < 0.001). The resulting non-perfused volumes from the GA experiments measured 0.70 ml (10 therapeutic sonications) and 0.51 ml (9 therapeutic sonications). The NPVs from the PSA experiments measured 0.70 ml (7 therapeutic sonications) and 1.16 ml (8 therapeutic sonications). The average NPV per sonication was 0.06 for the GA experiments and 0.21 for the PSA experiments. In conclusion, respiratory-gated MR-HIFU in the liver under PSA with spontaneous breathing is feasible and allows for a duty cycle which is at least comparable to GA. Remifentanil seems particularly suited for this purpose because of its strong inhibitory effect on the ventilation, which leads to a long resting phase of the diaphragm. A downside is the risk of apneas, which may require intervention (e.g. short-term ventilation of the patient). Future work will need to compare the frequency of patient movement under GA and PSA before clinical use is warranted.

